# Seasonal Effects on Body Condition and Characteristics of the Estrous Cycle in Captive Asian Elephants (*Elephas maximus*) in Thailand: A Retrospective Study

**DOI:** 10.3390/ani13071133

**Published:** 2023-03-23

**Authors:** Yuqing Yang, Padet Tummaruk, Taweepoke Angkawanish, Warangkhana Langkaphin, Kaywalee Chatdarong

**Affiliations:** 1 Research Unit of Obstetrics and Reproduction in Animals, Department of Obstetrics, Gynaecology and Reproduction, Faculty of Veterinary Science, Chulalongkorn University, Bangkok 10330, Thailand; 2 The Thai Elephant Conservation Center, National Elephant Institute of Thailand, The Forest Industry Organization, Lampang 52190, Thailand

**Keywords:** Asian elephants, body condition score, estrous cycle, overweight, progesterone, season

## Abstract

**Simple Summary:**

Thailand is in the tropics, with high temperatures, humidity, and long daylight hours. Seasons affecting estrous cycles have been discovered in the semi-captive Asian elephant in Thailand. In addition, seasonality in reproduction has been reported in sows and cows in the tropic environment. Despite the fact that measurements of the body condition score (BCS) have served as a disease diagnostic tool in terms of screening undernourishment and obesity in veterinarian practice and have been developed and applied in captive elephants, obesity is a growing concern today. The consequences of being overweight are typically not recognized until welfare-related reproductive dysfunctions, such as higher neonate weight, stillbirth, and acyclicity, are present. In captive-managed elephants, ovarian dysfunction and estrous cycling abnormalities are common, which are linked to a variety of issues such as obesity, reproductive tract pathologies, and metabolic derangements. These problems are most likely to contribute to poor reproductive health and then lead to undesirable reproduction. Thus, it is critical and urgent to identify the factors affecting reproductive health and ovarian patterns and thus facilitate fertility. The data for this study were collected from eight captive female elephants. During the rainy, hot, and cool seasons, their BCS was measured. Characteristics of the estrous cycle were studied, including the length of the estrous cycle and the lengths and starts of the luteal phase and follicular phase. The findings revealed that a higher BCS and the start of the follicular phase were highly frequent during the rainy season, which resulted in a longer luteal phase and estrous cycle and higher peak progesterone levels in the Asian elephant (*Elephas maximus*).

**Abstract:**

The aim of this study was to investigate the effects of season on the body condition score (BCS), the characteristics of the estrous cycle (luteal phase [LPL], follicular phase [FPL], estrous cycle [ECL] lengths, and the start of the luteal phase [SLP] and follicular phase [SFP]), and progesterone levels (baseline and peak) of eight captive Asian elephants (*Elephas maximus*) in Thailand. From 2014 to 2019, blood samples were collected weekly for serum progesterone enzyme immunoassays (EIAs). Estrous cycles (*n* = 70), including the luteal and follicular phases, and BCS (*n* = 70) were recorded. Based on the BCS, the LPL, FPL, and ECL were assigned to the following two groups: normal (BCS = 3.0–4.0, *n* = 38) and overweight (BCS = 4.5–5.0, *n* = 32). The findings demonstrated that there was no difference in LPL between the groups. However, in the normal group, the ECL was one week longer (14.9 ± 1.7 vs. 13.9 ± 1.7 weeks; *p* < 0.05), and the FPL also tended to be one week longer (7.2 ± 1.7 vs. 6.4 ± 1.5 weeks; *p* = 0.06) than in the overweight group. The mean progesterone level during the rainy, hot, and cool seasons was not statistically different. Based on the yearly averaged BCS from three seasons, the baseline and peak levels of progesterone were classified into the normal (*n* = 16) and overweight (*n* = 12) groups. Females with a normal BCS tended to exhibit higher progesterone peak levels (*p* = 0.08). The majority of peaks appeared during the rainy season (53.57%). The BCS was highest during the hot (4.47) and rainy (4.38) seasons, but not during the cool (4.12) season. The LPL, FPL, and ECL were not affected by the season in which the luteal phase occurred. On the other hand, the rainy season had a significant effect on the SFP, resulting in a longer LPL (*p* < 0.05) and ECL (*p* = 0.01); both were the longest during the rainy season. In conclusion, the effects of season on BCS may be related to characteristics of the estrous cycle and peak progesterone levels. Ultimately, these findings provide ground knowledge to assist elephant managers and owners in planning breeding activities using seasonal effects and BCS measurements in tropical climates.

## 1. Introduction

Elephants have been identified as an important flagship and keystone species. Maintaining elephants in self-sustaining populations is critical for the conservation of biodiversity and ecological balance. It is worth noting that the use of elephants has shifted from labor to tourism [1], which brings significant economic and cultural benefits to Thailand today. Regardless of their significance, Asian elephants (*Elephas maximus*) have been listed in Appendix I of the Convention on International Trade in Endangered Species of Wild Fauna and Flora (CITES) since 1975. Although there has been an 11.9% increase in the number of zoo-managed female elephants in Europe over the last four decades [2], a major issue in Asian countries is that captive populations are not self-sustaining due to a low birth rate, high mortality caused by an imbalanced sex ratio, a high proportion of non-reproductive-age females [1,3], undesirable semen quality in males [4,5,6], and, more importantly, unsolved reproductive problems of both sexes, etc. Ovarian and cycling abnormalities are common dysfunctions in captive female elephants [7,8], which are linked to a variety of issues such as obesity [9,10], reproductive tract pathologies [7], metabolic derangements [11,12], etc. These issues are most likely to contribute to poor reproductive health and then lead to undesirable fertility.

Thailand is in the tropics, with high temperatures, humidity, and long daylight hours. Seasons affecting estrous cycles have been discovered in Thai semi-captive Asian elephants [13]. Seasonality in reproduction has been reported in Landrace and Yorkshire sows [14] and lactating Holstein cows [15] in Thailand. Furthermore, the higher body condition score (BCS) of captive elephants in Thailand has been observed during the winter and rainy seasons [16,17], whereas the highest frequency of a lower BCS from elephants roaming free in India was observed during the dry season as opposed to the wet season [18]. Although the BCS has served as a disease diagnostic tool in terms of screening undernourishment and obesity in veterinarian practice, the management of overweight/obese elephants in captivity has faced challenges and difficulties. In Thailand, up to 39% of elephants have the highest BCS [16]. In addition, 74% of African and Asian elephants in North America are overweight [19]. BCS is a stress marker in cows [20], dogs [21], and goats [22], as well as Asian elephants [23,24,25], and has been identified as one of the gold standards indicating the welfare of zoo elephants [17,24,26]. In addition, monitoring the BCS significantly helps in determining reproductive performance [27] and ovarian dysfunction [28]. In elephants, the consequences of being overweight are typically not recognized until welfare-related reproductive dysfunctions, such as higher neonate weight [2], stillbirth [29,30], and acyclicity [10,12], are present. In addition, a high BCS is associated with abnormal ovarian cyclicity in African elephants [19].

Captive breeding management is critical for preserving genetic diversity in the face of restrictions on the movement of endangered species across borders. Asian and African elephants are thought to live for more than 70 years [31,32], and most females can continue to reproduce until the age of 60 [27]. Given these considerations, throughout the long lives of female elephants, the regular monitoring of blood progesterone is an important tool in the strategies and management of breeding in order to (1) monitor gestation to avoid stillbirth and predict the delivery time and ovarian cycle patterns [33]; (2) avoid unexpected pregnancies around the early puberty period [34]; (3) determine the optimal time for mating and control the management of females of prime breeding age (before 30 years of age) and old females management [8,35]; (4) diagnose pseudopregnancy by identifying the prolonged duration of the luteal phase [34], etc. Furthermore, determining whether elephants have a normal ovarian cycling status requires managers to know and calculate the baseline progesterone. Nevertheless, the understanding of the factors influencing the estrous cycle in this endangered species is limited.

The purpose of the present study was to gain a deeper understanding of the seasonal effects on the estrous cycle and determine if there are any relationships among BCS, seasons, progesterone levels (baseline and peak), and the characteristics of the estrus cycle (luteal phase [LPL], follicular phase [FPL], estrous cycle length [ECL], and the start of the luteal phase [SLP] and follicular phase [SFP]) in Asian elephants in Thailand.

## 2. Materials and Methods

### 2.1. Study Animals and Data Collection

This study was part of routine management at the National Elephant Institute (NEI), Forest Industry Organization, Lampang, Thailand (latitude 18°21.60′ N and longitude 99°14.92′ E).

Data were collected from January 2014 to December 2019. A total of 8 healthy females (E1 to E8) ranging in age from 9 to 41 years with a mean body weight of 3273 ± 515 kg (ranging from 2060 to 4080 kg) housed at the NEI were included (Table 1). The data of E4 were only obtained in 2019 because this female was suspected to be pregnant since 2020.

During the daytime, all females worked for tourist activities, either riding (with a saddle) or performing for no more than 4 h (not consecutively from 08:00 to 15:00). When not working, elephants were fed with Napier grass (*Pennisetum purpureum*), Pangola grass (*Digitaria eriantha*), Tamarind (*Tamarindus indica*), corn, carrot, and other natural foods. After working, all females were chained separately with long 20–30 m chains in the forest for foraging (from 16:00 to 18:30); the availability of foods varied with the seasons in the foraging area. The females were kept in separate locations from the males at night. Three times a year, veterinarians performed routine health examinations.

### 2.2. Sampling and Serum Progesterone Assay

All females were well-trained for blood collection. Blood samples were taken from the elephants’ ear veins weekly by veterinarians in the morning. Subsequently, the blood was clotted at room temperature and centrifuged at 2000× *g* for 10 min to obtain serum. Serum samples were stored at −20 °C until analysis.

Enzyme immunoassays (EIAs) were used to determine the concentrations of progesterone in the serum following previous protocols [13,36]. Briefly, serum progesterone concentrations were determined by monoclonal progesterone antibody assays (1:10,000; CL425, Coralie Munro, University of California, Davis, CA, USA), horseradish-peroxidase conjugated progesterone labeling (1:40,000), and progesterone standards (Catalog #P0130, Sigma Chemical Co., St. Louis, MO, USA). The antibody can cross-react with several progesterone metabolites in the elephant [37]. The sensitivity of the assay was 0.031 ng/mL. The intra- and inter-assay coefficients of variation were < 10%.

### 2.3. Calculations and Definitions

The calculation of the progesterone baseline was modified from previous reports [38,39]. Briefly, values greater than the mean plus 1.5 times the standard deviation (SD) were excluded until no more values exceeding this range were found. The remaining data were defined as the baseline. In a previous study, serum progesterone concentrations from Asian elephants ranging from 0.94 to 4.71 ng/mL (>3.99 ng/mL) were detected by using the same EIA protocol [34]. However, the maximum detectable value of EIA in this study was 3.99 ng/mL. Thus, data with a mean plus 1.5 times the SD of more than 3.99 were excluded when calculating the baseline progesterone. Furthermore, a progesterone concentration of 3.99 ng/mL was considered the peak during each luteal phase. The baseline and peak progesterone for each cycle of each individual elephant were first assessed and then averaged yearly.

The lengths of the luteal phase (LPL), follicular phase (FPL), and estrous cycle (ECL) were determined as previously described [38]. The first point at which the level of serum progesterone was greater than the baseline in the luteal phase and less than the baseline in the follicular phase was defined as the start of the luteal (SLP) and follicular (SFP) phases, respectively.

Monthly data regarding the average temperature and humidity were obtained from the Northern Meteorological Center, Meteorological Department, Ministry of Information and Communication Technology, Chiang Mai, Thailand, from 2014 to 2019. According to Thailand’s official seasonal time, three seasons were identified: the hot season (16 February to 15 May), the rainy season (16 May to 15 October), and the cool season (16 October to 15 February). The average humidity and temperature of the hot, rainy and cool seasons were estimated as 62.2% (range: 42.7–83.1%) and 29.0 °C (27.0–32.0 °C), 75.7% (23.7–91.5%) and 26.5 °C (22.4–30.5 °C) and 62.6% (23.2–75.1%) and 23.6 °C (19.7–26.8 °C), respectively.

### 2.4. Body Condition Scores

The body condition score (BCS) assessment method was modified from a previous study in which an 11-point scale BCS system was developed for the Asian elephant [40]. For more than a decade, experienced veterinarians in the NEI have converted the 11-point scale to a 5-point scale (1–5 from thinnest to fattest), based on visual observations of the same body regions using the same criteria: (1) head (0–0.5 points)—multiple-angle views to determine the temporal depression; (2) scapula (0–1.0 points)—lateral views to determine the visibility of the spinous process; (3) thoracic region (0–1.0 points)—lateral views to determine the visibility of the ribs; (4) the flank area (the region in front of the pelvic bone, 0–0.5 points)—lateral and rear views for depression in this area; (5) lumbar vertebrae (behind the ribs and in front of the pelvis, 0–1.0 points)—rear views to determine the visibility of the lumbar vertebrae; and (6) the pelvic bone (0–1.0 point)—multiple-angle views to determine the visibility of the pelvic bones. The points from each observation region were added. Elephant BCS with a single-digit score greater than 5 was added to the higher score group; otherwise, BCS was placed in the lower-score category. BCS was measured once each at the beginning of the rainy, hot, and cool seasons by three well-trained and experienced veterinarians throughout the whole year. Finally, BCS was averaged from three measurements.

### 2.5. Experimental Design and Statistical Analysis

All statistical analyses were performed using Statistical Analysis System version 9.4 (SAS Institute, Cary, NC, USA). Spearman’s correlation coefficient (*r*) was used to determine the associations among the lengths of the luteal phase (LPL, *n* = 70), follicular phase (FPL, *n* = 70), and estrous cycle (ECL, *n* = 70). Non-parametric Kruskal–Wallis tests were performed to find the effects of the seasons (rainy, hot, and cool seasons) on the SLP and SFP. Based on the BCS, the LPL, FPL, and ECL were assigned to the normal (BCS = 3.0–4.0, *n* = 38) and overweight groups (BCS = 4.5–5.0, *n* = 32). A general linear model (GLM) was used to detect (1) differences in the LPL, FPL and ECL when the SLP and SFP occurred over three seasons, including the start of the luteal and follicular phases as fixed effects and individual elephants as random effects; (2) differences in the LPL, FPL, and ECL between the normal and overweight groups, including the mean level of the two groups as fixed effects and individual elephants as random effects. One-way Analysis of Variance (ANOVA) was performed to determine seasonal differences in the mean progesterone concentration. Moreover, BCS was averaged yearly from three seasons of data. Subsequently, according to the BCS, baseline and peak progesterone concentrations were also assigned to the normal (*n* = 16) and overweight (*n* = 12) groups. Student’s *t*-test was performed to determine differences in baseline and peak progesterone between the two groups. The PROC MIXED procedure of SAS was performed to determine the differences among seasons. The statistical models included the years of data collection (2014–2019), seasons, and all appropriate interactions as fixed effects, and elephant ID as a random effect.

For all models, least square means were used when significance was indicated (*p* < 0.05). All data are presented as the mean ± SD. All statistical differences were set at *p* < 0.05 with a confidence interval of 95%.

## 3. Results

In total, 70 complete estrous cycles comprising the luteal and follicular phases were analyzed. Table 1 shows the number of estrous cycles obtained from each elephant as well as the background information. The females were either primiparous (*n* = 5) or nulliparous (*n* = 3). For 2014, 2015, 2016, 2017, 2018, and 2019, the mean (±SD) ECL, LPL, and FPL of all elephant females were 14.5 ± 1.8, 7.6 ± 1.6 and 6.8 ± 1.7 weeks, respectively. ECL was found to be positively (*p* < 0.001) correlated with both FPL (*r* = 0.57) and LPL (*r* = 0.51). Additionally, LPL was negatively correlated with FPL (*r* = −0.35, *p* = 0.003).

No seasonal effects on the SLP (*χ^2^* = 2.76, *df* = 2, *n* = 70) and SFP (*χ^2^* = 0.32, *df* = 2, *n* = 70) were found (*p* > 0.05). Normal elephants had a longer ECL than overweight elephants [14.9 ± 1.7 weeks (range = 11–18 weeks) vs. 13.9 ± 1.7 weeks (range = 10–17 weeks); *p* = 0.02] (Table 2). The FPL for females in the normal (7.2 ± 1.7, 4–11 weeks) group tended to be longer (*p* = 0.06) than those in the overweight (6.4 ± 1.5, 3–9 weeks) group. LPL, on the other hand, did not differ between these two groups (*p* > 0.05). The coefficient of variation (CV) for LPL, FPL, and ECL was 19.3%, 24.2% and 11.6%, respectively, for the normal group and 22.7%, 23.7%, and 12.1%, respectively, for the overweight group.

The overall baseline and peak concentrations of progesterone for the elephants were 0.93 ± 0.3 ng/mL (range, 0.45–1.33) and 3.77 ± 0.4 ng/mL (range, 2.58–3.99 ng/mL), respectively. The peak in progesterone was most frequently observed during the rainy season (53.6%, *n* = 15), followed by the cool (25.0%, *n* = 7) and hot seasons (21.4%; *n* = 6). The mean progesterone levels in the hot, rainy, and cool seasons were not statistically different (1.16 ± 0.3, 1.21 ± 0.4, and 1.11 ± 0.4 ng/mL). Although the baseline concentrations of progesterone were not different between the two groups (*p* > 0.05), the peak progesterone concentration (3.91 ± 0.3 and 3.59 ± 0.5 ng/mL, respectively) of the elephants with a normal BCS tended to be higher (*p* = 0.08, Table 3).

Female elephants had an overall mean BCS of 4.26. Season had a significant impact on BCS (*p* < 0.05). Elephant BCSs obtained during the cool season were lower than those in the rainy (mean ± SEM, 4.12 ± 0.15 vs. 4.38 ± 0.15, *p* = 0.04) and hot seasons (4.47 ± 0.16, *p* = 0.02). However, the BCSs obtained during the rainy and hot seasons were not different (*p* > 0.05). The most prevalent BCS was 4.0 (41.4%, *n* = 29), followed by 5.0 (24.3%, *n* = 17), 4.5 (21.4%, *n* = 15), 3.5 (8.6%, *n* = 6), and 3.0 (4.3%, *n* = 3). In this study, none of the elephants had a BCS less than 3.0.

The rainy season had the highest frequencies of the luteal (38.57%, *n* = 27) and follicular (40.0%, *n* = 28) phase, while the cool season had the lowest frequencies of both phases (28.57%, *n* = 20 and 27.14%, *n* = 19, respectively). The number of luteal and follicular phases was similar (32.86%, *n* = 23) during the hot season. When the SFP occurred during the rainy season, the LPL (*p* = 0.04) and ECL (*p* = 0.01) were significantly longer (Table 4). During the hot and cool seasons, neither SFP nor SLP resulted in any differences in LPL, FPL, and ECL.

## 4. Discussion

The female elephants with a normal BCS had an ECL that was one week longer and an FPL that was nearly one week longer, which were in the normal range. Compared with their normal counterparts, the overweight females in this study had higher variability in the ranges of their ECL and LPL. Nutritional status has a direct impact on BCS. It has been proposed that mares and goats with abnormally low and high BCSs have abnormal estrous cycle lengths [41,42]. In addition, in Creole goats, a temporal restriction in feeding contributes to the increased length of the anovulatory period [42]. Likewise, indigenous central African cattle fed with a low-plane diet have longer estrous cycles [43]. Even though high variability in the characteristics of the estrous cycle across or within individuals was found, the overall LPL, FPL, and ECL were within the range of the previous data [39,44]. Moreover, the present findings agreed with previous reports that the follicular phase is much shorter than the luteal phase in a complete estrous cycle in Asian elephants [13,39]. Although the scales and systems used to estimate the BCS differ among countries and even cities, low indexes represent emaciation and high indexes indicate obesity. Visual body condition scores, which are based on the visibility of the skeletal structures, provide an indirect measurement of fatness in companion animals [45], livestock [46,47], marine animals [48], and elephants [49]. A high-energy diet containing high-calorie and high-sugar fruits while training and participating in tourist activities is likely to result in a higher BCS in elephants in Thailand [50]. Measurement and management of the BCS can help elephant owners and managers become more aware of changes in feeding, such as optimizing the quality and quantity of food, which can reduce the likelihood of obesity and even abnormal estrous cycle length.

The female elephants had a higher BCS during the hot and rainy seasons, which was inconsistent with previous studies indicating that the BCS was higher during the peak tourist season (cool season) and hot season in Thailand [16,17]. Wet seasons create an environment with a higher distribution of water resources and greater availability of plants and grass in the foraging area, both of which contribute to a higher BCS or body weight in semi-captive and free-ranging elephants [18,24,51]. On the other hand, elephants in captivity are fed sufficient food all year. Routine management in the NEI primarily consists of tourist activities and foraging in the forest. The chief elephant keepers in the NEI have optimized and regulated the daily food allowance by only giving each elephant 6.0% of their body weight in food. It is also important to note that the administrators have attempted to incorporate low-calorie foods such as corn and carrots into the routine feeding supplements and visitor activities, particularly for elephants that are overweight. In addition, the feeding protocol remains consistent during working hours across seasons in the NEI. As a result, these differences are most likely due to the availability of seasonally natural foods and plants in foraging areas. Aside from the environment, obesity is associated with genes such as leptin and contributes to overweight conditions in cattle [52], pigs [53], and African elephants (*Loxodonta africana*) [54].

The present findings show that the rainy season affects the SFP, thereby significantly increasing the LPL and ECL in female elephants. Captive elephants do not obligatorily breed seasonally and have ovarian cycles throughout the year. The temperature–humidity index (THI) in northern Thailand is higher during the late summer and early rainy seasons. A comprehensive review found that daylight length increases in Thailand from January to June, and elephants are likely to adjust their cycles according to the photoperiod of the seasons, which is associated with photoperiodicity and always coincides with seasonal triggers such as the wet seasons and high BCS in the tropic and subtropics [55]. However, the season did not affect the serum concentration of prolactin because captive elephants are exposed to artificial lighting, unlike wild elephants housed outdoors all time, indicating that elephants in captivity may adjust their cycles according to the non-natural light instead of the seasonal effects [56]. The current data agreed with a previous study that reported that the follicular phase plays a critical role in regulating and maintaining the overall duration of the estrous cycle in Asian elephants; this is because there are many more variables in this status than in the luteal phase [39]. Double luteinizing hormone (LH) surges have been discovered to be a distinctive feature of Asian elephants [57,58,59]. Only the largest follicle of the first follicular wave, known as the accessory corpus luteum (acCL), is luteinized, while small follicles regress after the first surge in LH. A second LH peak leads to ovulation after a three-week interval because the dominant follicle’s ovulatory CL (ovCL) of the second follicular wave produces the largest CL [57,60,61]. As previously reported, this interval in the follicular phase is longer during the rainy season, most likely due to this season having the greatest temperature, humidity, and daylight length throughout the year [13]. In this study, the SFP occurred most during the rainy season (40%). Follicle growth is sensitive to THI. Under field conditions, an increase in the THI is associated with rising progesterone and a reduction in the size of follicles in cows [62]. Likewise, in an in vivo study of cattle, higher temperatures increased the secretion of progesterone and induced the follicular cells to luteinize [63]. In this regard, it is not surprising that the progesterone levels of elephants in this study remained high and frequently peaked during the rainy season, given that elephants also have a higher fecal concentration of 5-pregnane-3-ol-20-one during wet seasons, which is driven by the higher THI [51]. However, the mechanism of how nutrition affects follicular growth in elephants needs more investigation.

Moreover, the availability of seasonal foods could be responsible for the onset of the breeding period. In the tropics, mammals are more likely to start their reproductive cycles and peak conception rate by meeting their metabolic requirements such as eating large amounts of high-quality food [64,65]. In the elephant, normal cycling is inconsistent, and the causes of disruptions to and dysfunctions of the ovarian cycle are not entirely clear. Leptin can directly modulate ovary activity [66]. Insulin stimulates ovarian actions such as steroidogenesis via the insulin receptor or insulin-like growth factor 1 (IGF-1) receptor [67]. Plenty of evidence has shown that a high BCS may be an indicator of estrous abnormality and metabolic derangements in elephants. Specifically, high BCS is associated with non-ovarian cycling status in African elephants [19]. The glucose-to-insulin ratio (G: I) is inversely related to the BCS of elephants [11,12,16,17]. In addition, leptin [11,12] and insulin [12] levels are positively associated with non-cycling elephants, and the BCSs of elephants in the normal cycling group are lower, suggesting that being obese or overweight may threaten reproductive function. A recent study proposed that, while the mechanism of metabolic function in the elephant is unknown, the metabolic imbalance is not solely related to the level of body fat, most likely manipulating ovarian activity in overweight elephants [12]. Furthermore, obese horses have higher blood cortisol levels [68]. In Asian elephants, glucocorticoids (GC) have been recognized as a stress indicator that is positively related to BCS [18,25] and associated with the peak tourist season (a higher number of tourists) [17]. Controlling mammals with a normal or optimal BCS before mating increases the likelihood of pregnancy [69,70]. In addition, females with a normal or ideal BCS have better reproductive performance and fertility rates [71,72,73]. The highest success rate of conception in elephants has been identified in the rainy season in Thai tourist camps [3]. Although no sole effective breeding strategy for captive elephants has been developed, the monitoring of ovulation and the standard BCS is recommended during the rainy season. Moreover, predicting the optimal timing for natural mating or artificial insemination (AI) could help improve fertility.

This retrospective study has implications for improving the management strategies and welfare of elephants. It is critical to remind elephant managers to choose foraging areas with a high proportion of roughage or high-fiber foods, rather than energy-rich foods, for overweight elephants. The quantity of food provided to elephants participating in tourist activities is sometimes excessive, and the quality of foods, particularly low-sugar and low-calorie foods, should be considered. Attempts have been previously made in the past to address elephant obesity. For example, unpredictable feeding schedules and more staff-directed walking exercises significantly reduced the risks of high BCS in zoo-managed elephants in North America [74]. Increased walking duration (7 h or more per week), random feeding schedules, and more available space have recently been proposed in Thailand to address ovarian acyclicity and obesity [64]. Longer distances of walking or riding (saddle or bareback) under human control prevent boredom and contribute to a better BCS, healthy adrenal activity, and metabolic status [16,75], ultimately optimizing elephants’ welfare. These management strategies, on the other hand, are not unified standards and are probably not applicable to every facility. Elephant managers are supposed to set a financial budget and reality-based standards.

## 5. Conclusions

Seasonal effects on the BCS and characteristics of the estrous cycle in captive-managed female Asian elephants in tropical environments were found. In addition, the current findings suggested that a higher BCS and the start of the follicular phase were the most frequent during the rainy season, lengthening the luteal phase and estrous cycle of the elephants. To reduce the risks of an abnormal estrous cycle length, management strategies such as maintaining elephants in normal body condition by promoting proper exercise and adjusting their feeding schedules are beneficial. In addition, elephant managers should plan breeding activities based on seasonal effects and BCS measurements in order to maximize reproduction rate and animal welfare. However, the relationships among metabolic/ovarian and health parameters such as IGF-1, insulin, and leptin; feeding schemes; follicular growth; and characteristics of the estrous cycle in elephants would be necessary and interesting to discover.

## Figures and Tables

**Table 1:** Data recorded during the study period and background information on the female elephants (*n* = 8) housed at the National Elephant Institute (NEI).

Elephant	Years of Data Collection (Number of Cycles)	Age during Study Period (y)	Reproductive History	Type of Work	Total Number of Cycles
E1	2015 (2), 2016 (1), 2017 (4), 2018 (4), 2019 (3)	37–41	Primiparous	Riding	14
E2	2014 (2), 2017 (3), 2018 (4), 2019 (3)	19–24	Primiparous	Riding	12
E3	2016 (1), 2017 (4), 2018 (4), 2019 (3)	34–37	Primiparous	Show	12
E4	2015 (2), 2017 (3), 2018 (3), 2019 (3)	17–21	Primiparous	Show	11
E5	2019 (2)	9	Primiparous	Show	2
E6	2015 (1), 2017 (1), 2018 (1)	25–29	Nulliparous	Riding	3
E7	2015 (2), 2016 (1), 2017 (3), 2018 (2), 2019 (2)	10–14	Nulliparous	Show	10
E8	2017 (3), 2018 (3)	36–37	Nulliparous	Show	6

**Table 2:** Comparison of LPL, FPL and ECL (weeks) between the normal and overweight groups in Asian elephants (*n* = 8).

	Normal (*n* = 38)	Overweight (*n* = 32)	*p*-Value
LPL	7.7 ± 1.5	7.5 ± 1.7	0.58
	(5–13)	(5–11)	
FPL	7.2 ± 1.7	6.4 ± 1.5	0.06
	(4–11)	(3–9)	
ECL	14.9 ± 1.7	13.9 ± 1.7	0.02 *
	(11–18)	(10–17)	

Abbreviations: LPL, luteal phase length; FPL, follicular phase length; ECL, estrous cycle length; *n*, number of phases or cycles. Data are presented as the mean ± SD and range (min–max). * *p* < 0.05.

**Table 3:** Comparison of peak and baseline concentrations of progesterone (ng/mL) between normal and overweight groups of Asian elephants (*n* = 8).

Progesterone Concentration	Normal	Overweight	*p*-Value
	(*n* = 16)	(*n* = 12)	
Peak	3.91 ± 0.3	3.59 ± 0.5	0.08
	(2.97–3.99)	(2.58–3.99)	
Baseline	0.98 ± 0.3	0.87 ± 0.2	0.29
	(0.45–1.33)	(0.56–1.29)	

Data are presented as the mean ± SD and range (min–max).

**Table 4:** Seasonal effects of SLP and SFP and their influence on LPL, FPL and ECL (weeks) in Asian elephants (*n* = 8).

Season	SLP				SFP			
	*n*	LPL	FPL	ECL	*n*	LPL	FPL	ECL
Cool	20	7.5 ± 1.6	6.5 ± 1.7	14.0 ± 1.8	19	7.7 ± 1.5 ^ab^	6.6 ± 1.6	14.4 ± 1.8 ^ab^
Hot	23	7.7 ± 1.9	6.7 ± 1.6	14.3 ± 1.8	23	7.0 ± 1.5 ^a^	6.7 ± 1.6	13.7 ± 1.6 ^a^
Rainy	27	7.7 ± 1.3	7.3 ± 1.7	15.0 ± 1.7	28	8.1 ± 1.6 ^b^	7.1 ± 1.8	15.1 ± 1.7 ^b^
*p*-Value		0.86	0.25	0.13		0.04 *	0.64	0.01 *

Abbreviations: LPL, luteal phase length; FPL, follicular phase length; ECL, estrous cycle length; SLP, start of the luteal phase; SFP, start of the follicular phase; *n*, number of phases or cycles. Data presented as mean ± SD. Values in the same column with different superscript letters differ significantly (*p* < 0.05). * *p* < 0.05.

## Data Availability

Not applicable.
